# In their narratives: academic socialization in the experience of sensory processing sensitivity among university students

**DOI:** 10.3389/fpsyg.2024.1448443

**Published:** 2024-10-23

**Authors:** Marzia Saglietti, Mara Marini, Stefano Livi

**Affiliations:** ^1^Department of Social and Developmental Psychology, Sapienza University of Rome, Rome, Italy; ^2^Department of Neuroscience, Imaging and Clinical Sciences, University of Chieti-Pescara, Chieti, Italy

**Keywords:** sensory processing sensitivity, university students, academic socialization, interviews, teacher–student relationship, peer relationship, thematic analysis, environmental sensitivity

## Abstract

**Introduction:**

While much of the worldwide contemporary research on sensory processing sensitivity (SPS) and environmental sensitivity (ES) has relied on the participation of university students, there remains a significant gap in understanding the academic social experiences of those scoring high in SPS (i.e., highly sensitive individuals).

**Methods:**

To address this gap, this exploratory study aimed to investigate in detail students’ academic socialization through their narratives. We conducted nine interviews with Italian university students who self-identified as highly sensitive.

**Results:**

Through thematic reflexive analysis, we identified and analyzed 6 themes (with subthemes and versions of subthemes) concerning their self-definitions, their university experience (in classroom, before, during, and after exams), and socialization with peers and teachers.

**Discussion:**

After 20 years of research on SPS, this study integrates the relevant literature into the field of social psychology and academic socialization, emphasizing the importance of understanding SPS within real-life educational contexts and considering highly sensitive students’ perspectives on their resources and challenges in attending university. By contributing to the emerging qualitative literature on SPS and ES, this study provides practical implications for educators and policymakers seeking to foster inclusive learning environments for all students.

## 1 Introduction

Most contemporary research on adult sensitivity is owed to university students—especially those studying psychology. From the earliest investigations (Aron and Aron, 1997; Aron et al., 2005; Jagiellowicz et al., 2011; Liss et al., 2005, 2008) onwards, most experimental tasks, ranging from visual experiments to brain scanning, personality tests, and social surveys concerning sensory processing sensitivity (SPS) and environmental sensitivity (ES; see Pluess, 2015; Greven et al., 2019) have been mainly based on the involvement of this specific population. Yet, the perspectives of university students scoring high in SPS—broadly speaking, highly sensitive university students—remain largely underexplored. Particularly, we know very little about their academic socialization, i.e., the socialization process in which students, on entering the university system, are exposed to a wide range of socializing influences from both internal groups (peers, teachers, staff) and external groups outside the university (parents, friends, etc.), affecting their goals, values, and academic or professional aspirations (Weidman et al., 2014). Concerning highly sensitive students, academic socialization largely remains underexplored. Thus, we do not know what the perceived impact of SPS is on their academic performance and broader socialization, nor what their challenges and resources with either peers or teachers are. Filling this gap and building on these considerations, in this article we focus on highly sensitive university students’ narratives on academic socialization.

## 2 Academic socialization

To successfully navigate university environments, students must learn new rules, regulations, and implicit norms of their universities, acquire new learning skills and strategies, and actively participate in the social dynamics of academia (Farnese et al., 2022). This process, i.e., academic socialization, inevitably poses various stressors for young students, as it may lead to changes in lifestyle, interpersonal relationships, and mental health (Yano et al., 2021).

For many years, academic socialization has been studied through an individualistic lens. Students’ adaptation to university systems was attributed primarily to personal factors (e.g., attributes, skills, motivation)—a perspective now recognized as placing undue blame on students (Tinto, 1975, 2006, 2022). However, academic socialization processes are socially situated. According to recent theoretical models (Weidman, 2006; Weidman et al., 2014), students’ adaptation to university is an ongoing, socially iterative process influenced by both individual characteristics and characteristics of university environments (Weidman, 2006; Weidman et al., 2014). In this light, to understand students’ experiences at university, it is important to account for the unique characteristics of students and the wide range of experiences to which they are exposed. Considering that students’ success is crucial for the socio-economic well-being of our societies (European Commission, 2017), improving students’ university experience is pivotal for educational institutions to reduce the dropout rate and promote students’ well-being. In Italy, where this study was conducted, despite recent improvements, students’ success at university is still a cause for concern, also after the difficulties caused by the COVID-19 pandemic (AlmaLaurea, 2023). Despite this, research with university students, especially those from at-risk social groups and minority groups—those more prone to university stress (Aschieri et al., 2024)—remains limited, and has focused mainly on distal outcomes and related factors rather than giving voice to minority students’ experiences.

### 2.1 The academic socialization of highly sensitive students

Highly sensitive students are estimated to make up around 15–20% of the entire population, and this percentage is believed to be even higher among those studying psychology (Aron and Aron, 1997), who have been extensively involved in much of the current research on SPS and ES. Over the years, this scholarship has shown that students with high scores on SPS are at great risk of maladaptive behaviors and negative developmental outcomes (May and Pitman, 2023), ranging from mental health issues—mostly anxiety (Liss et al., 2005, 2008), depression (Liss et al., 2005), and alexithymia (Liss et al., 2008)—to overall lower subjective well-being, with stronger emotional activation and perceived stress (Gerstenberg, 2012; Jagiellowicz et al., 2016; Rubaltelli et al., 2018).

To our knowledge, only two studies have analyzed in depth the experiences of highly sensitive students at university. In their research involving 580 undergraduate South African psychology students, May and Pitman (2023) documented that those high in SPS reported significantly worse adjustment to university. According to the authors, this was due to students’ higher neural sensitivity and strong negative affectivity. Nevertheless, their more accurate depth of information processing and aesthetic sensitivity seemed to foster overall improved academic success. Similarly, in their study on a large cohort of highly sensitive Japanese university students, Yano et al. (2021) found a negative relationship between emotion-coping skills and depression specifically among high-SPS students. Hence, although these students tended to experience heightened negative emotions, robust emotion-coping abilities may mitigate the likelihood of experiencing significant depressive tendencies. Conversely, highly sensitive students tend to have enhanced decision-making skills. Astonishingly, however, less attention has been paid to their academic experiences. Bridging this gap, in this study we aimed at (1) understanding the broader academic experience of highly sensitive university students, focusing in particular on their academic socialization and (2) understanding what helps them and hinders them in academic socialization, whether per specific coping strategies, abilities, and/or situations.

## 3 Materials and methods

This study was part of an ongoing exploratory project on highly sensitive university students’ narratives. For this particular study, we relied on nine semi-structured interviews with highly sensitive Italian university students. Our research procedures were in line with ethical standards set by the Italian Psychological Association Code (AIP; 2015/2022) and the 1964 Helsinki Declaration. Respondent anonymity was assured, and participants, after being informed about the aims of the study, signed a consent form in line with all ethical procedures and GDPR norms active at the time.

### 3.1 Participants

#### 3.1.1 Recruitment

Participants were recruited on a voluntary basis by the interviewer. Eligibility criteria for participation included being a university student and being highly sensitive. To ensure this, participants were required to complete the 20-item “Are You Highly Sensitive?” self-test by Aron (1996) prior to participation. In line with Aron’s (1996, 1999) indications, those who positively responded to at least 12 items were considered eligible for participation. The sample size reached a minimum level of saturation (Hennink and Kaiser, 2022) due to the homogeneity of the participants (i.e., students who self-identified as highly sensitive) and the relatively narrow focus of the study (i.e., their academic experiences).

#### 3.1.2 Sample characteristics

Table 1 displays the main characteristics of the selected participants, listed with their pseudonyms.

**Table 1:** Participants’ characteristics (names are pseudonyms).

Interview #	Participant	Gender	Age	University	Degree in	Academic year
#1	Chiara	F	25	XXX_U	Clinical Psychology, MA	Fifth
#2	Francisca	F	21	XXX_B	Communication Science, BA	Fifth
#3	Giada	F	24	XXX_B	Pedagogy, MA	Fifth
#4	Aline	F	27	XXX_B	Communication Science, BA	Second
#5	Karola	F	24	XXX_B	Statistical Science, MA	Fifth
#6	Elisa	F	25	XXX_B	Pedagogy, MA	Fifth
#7	Anna	F	24	XXX_B	Architecture, MA	Fifth
#8	Giulio	M	24	XXX_U	Social Work, MA	Fifth
#9	Alessandra	F	24	XXX_B	Pedagogy, MA	Fifth

### 3.2 Data collection

Participants were interviewed using a semi-structured format (see Table A1 for the detailed list of questions). The interviews, each lasting about an hour, were conducted from March to July 2022. Two pilot interviews were used to test the structure of the interview and were thereafter included as part of the dataset. Interviews were conducted in Italian either via Zoom or face-to-face and video-recorded.

### 3.3 Data analysis

Video-recorded interviews were transcribed verbatim and analyzed with the reflexive pen-to-paper approach of thematic analysis (Patton, 1990; Braun and Clarke, 2006, 2022; Pagani, 2020). Following this approach, the authors familiarized themselves with the data through an initial exploration of the students’ narratives, consisting of an individual reading of the transcripts accompanied by note-taking. During the second reading of the transcripts, initial codes were generated on the basis of the aims of the study. These codes were revised by the first two authors and evolved not only on the basis of an ever-increasing knowledge of the text but also on the basis of comparison between researchers. The initial themes were then generated, and, again, MS and MM engaged in a critical discussion aimed at eliminating inconsistencies and outlining salient meanings that might be contained in a relevant theme. These initial themes were then revised according to the criteria of internal homogeneity and external heterogeneity. For each theme, the extracts coded in the previous phase were reread to ensure their consistency with the concept expressed in the theme; if they belonged to more than one theme, they were coded accordingly (i.e., twice or more than twice, if necessary, for different themes). Once the inconsistencies between codes and themes were resolved and an agreement on coding was reached, the themes were named and defined.

## 4 Results

Six main themes emerged from the coding: (1) self-definitions; (2) study approach, (3) classroom experience, (4) physical, emotional, and cognitive states during and after exams (or thesis discussion) (that for brevity we identified together as university experience); (5) peer relationships, and (6) student-teacher relationships. Each main theme was composed of themes, subthemes, and specific variants of subthemes. Tables 2–5 display the structure of each main theme; this text reports excerpts from participants pertaining to subthemes or variants of subthemes. They were translated into English by the authors.

**Table 2:** Self-definitions themes found in our analysis.

	Theme	Code	Sub-theme	Definition	Who talks about it	N total turns
A	**Self-definitions**	A1	As an anxious person	Interviewees describe themselves as anxious persons	I2, I3, I6, I8, I9	26
		A2	As a perfectionist person	Interviewees describe themselves as perfectionist persons	I2, I9	26
		A3	As a shy person	Interviewees describe themselves as shy persons	I1, I2, I3	10

**Table 3:** University experience themes and subthemes found in our analysis.

	**Theme**	**Code**	Sub-themes	**Definition**	**Who talks about it**	**N total turns**
B	**Study approach**	B1	**Strategies for organizing the study**	Interviewees describe their study approach	I3, I4, I5, I6, I8, I9	104
		B1.1	Challenging and tiring study approach	Interviewees evaluate their study approach as particularly tiring	I2, I3, I7, I8, I9	42
		B1.2	Excessive commitment	Interviewees evaluate their study approach as excessive	I2	11
		B2	**High school/university performances**	Interviewees refer to being satisfied with university grades and overall performance	I1, I2, I3, I4, I5, I6, I7, I9	65
		B3	**University career blocks**	Interviewees describe to have encountered some blocking experience in their academic career	I1, I4, I6	93
C	**Classroom experience**	C1	**Online class experiences**	Interviewees describe their evaluations and behavioral occurrences when taking online lessons	I2, I3, I4, I5, I6, I7, I8	155
		C1.1	Distractions in online lessons	Interviewees describe following online lessons a distracting activity	I2, I3, I5, I6, I7, I8	90
		C1.2	Preference for online lessons	Interviewees declare their preference for online lessons	I3, I4, I6	65
		C2	**Anxiety in speaking during lectures**	Interviewees narrate their fear of speaking in public at lesson	I1, I3, I4, I6, I7	38
D	**Physical, emotional and cognitive states during and after exams (or thesis discussion)**	D1	**Preference for written exams**	Interviewees express preference for written exams (instead or oral)	I1, I2, I3, I4, I5, I7	27
	D2	**Anxiety during exams**	Interviewees explicitly refer to anxiety issues related to the exam	I1, I2, I3, I4, I5, I6, I7, I8, I9	365
	D2.1	Exam anxiety	Interviewees describe when they felt anxious in the middle of an exam	I1, I2, I3, I4, I5, I6, I7, I8, I9	122
	D2.2	Technological anxiety during online exams	Interviewees describe when they felt anxious during an online exam due to technological reasons	I2, I3, I4, I6, I8, I9	66
	D2.3	Blocks during exams	Interviewees describes when they block themselves during an exam	I4, I5, I7, I8, I9	99
	D2.4	Being influenced by the presence of others	Interviewees describe when they felt anxious during an exam in relation to their being observed and judged by other (students)	I1, I2, I3, I4, I6, I7	51
	D3	**Physical symptoms in the post-exam (or post-thesis discussion) phase**	Interviewees narrate their physical sensations after taking the exam (or having discussed the thesis)	I2, I3, I4, I5, I6, I7, I8, I9	87
	D3.1	Physical tiredness	Interviewees narrate their being physically exhausted after taking an exam (or having discussed the thesis)	I2, I3, I4, I6, I9	29
	D3.2	Mental tiredness	Interviewees narrate their being mentally exhausted after taking an exam (or having discussed the thesis)	I2, I3, I4, I5, I8	32
	D3.3	Headache	Interviewees narrate their headache after taking an exam (or having discussed the thesis)	I5, I6	2
	D3.4	Leg problems	Interviewees describe their pain to the legs after taking an exam (or having discussed the thesis)	I3, I8	7
	D3.5	Neck pain	Interviewees describe their pain to the neck after taking an exam (or having discussed the thesis)	I8	2
	D3.6	Digestive problems	Interviewees describe their digestive problems after taking an exam (or having discussed the thesis)	I5	1
	D3.7	Hair loss	Interviewees describe their hair loss after taking an exam (or having discussed the thesis)	I9	6
	D3.8	Fever	Interviewees describe having fever after taking an exam (or having discussed the thesis)	I7	8
	D4	**Emotional experiences in the post-exam (or post-thesis discussion) phase**	Interviewees describe their emotions after taking an exam (or having discussed the thesis)	I1, I3, I4, I5, I6, I7, I8, I9	143
	D4.1	Relief	Interviewees describe relief after taking an exam (or discussed the thesis)	I1, I3, I4, I5, I6, I7, I8, I9	49
	D4.2	Contentment/Satisfaction	Interviewees describe their satisfaction after taking an exam (or discussed the thesis)	I1, I9	53
	D4.3	Disappointment/Guilt	Interviewees describe their disappointment after taking an exam (or discussed the thesis)	I1	1
	D4.4	Anger	Interviewees describe their anger after taking an exam (or discussed the thesis)	I9	39
	D4.5	Sadness	Interviewees describe their sadness after taking an exam (or discussed the thesis)	I1	1
	D5	**Cognitive-behavioral strategies in the post-exam (or post-thesis discussion) phase**	Interviewees describe their post-exams (or post-thesis) thoughts and strategies	I1, I2, I4, I5, I6, I8, I9	70
	D5.1	Distractions/Relax	Interviewees describe their need to relax after taking the exam (or having discussed the thesis)	I1, I2, I4, I5, I6, I8	30
	D5.2	Avoidance to contain anxiety	Interviewees describe their need to avoid talking of the exam to contain anxiety after taking the exam (or having discussed the thesis)	I5, I9	28
	D5.3	Brooding	Interviewees describe their brooding tendency after taking the exam (or having discussed the thesis)	I1	12

**Table 4:** Peer relationships themes and subthemes found in our analysis.


Theme	Code	Sub-themes		Sub-subthemes	Definition	Who talks about it	*N* total turns
E
	Peer relationships	E1	Peer relationships quality	E1.1	Difficult relationships	Interviewees describe their peer relationships as somehow difficult	I1, I2, I3, I6, I7	141
				E1.2	Positive relationships	Interviewees describe their peer relationships as somehow positive	I1, I3, I6 I7, I9	39
		E2	Barrier conditions	E2.1	Peer anxiety	Interviewees describe to feel social anxiety with reference to peer interaction	I3, I4, I6, I, I8, I9	104
				E2.2	Risk of self-exclusion	Interviewees describe their risk of isolating themselves from peers	I9	12
		E3	Favorable conditions	E3.1	Working in small groups	Interviewees describe working in small groups with colleagues as a positive condition	I1, I3, I4, I8, I9	73
E3.2				Importance of a peaceful environment	Interviewees describe being in peaceful social relationships as a positive condition	I1, I3, I5	40

**Table 5:** Student–teacher relationship themes and subthemes found in our analysis.


Theme	Code	Sub-themes		Sub-sub themes	Definition	Who talks about it	*N* total turns
F
	Student-teacher relationships	F1	Student-teacher relationships quality	F1.1	Difficult relationships	Interviewees describe their relationships with teachers as somehow difficult	I1, I3, I4, I5, I6, I7, I8, I9	181
				F1.2	Positive relationships	Interviewees describe their relationships with teachers as somehow positive	I2, I4, I5, I6, I7, I8, I9	150
		F2	Barrier conditions	F2.1	Struggles to enter into relationships/fear of judgment	Interviewees describe their struggles to be in good relationships with teachers	I1, I3, I5, I6, I7	76
		F3	Favorable conditions	F3.1	Need for calmness/reassurance/feedback	Interviewees describe and advise teachers to co-construct calm environments where reassurance and feedback are provided	I1, I2, I3, I4, I5, I6, I7, I8, I9	351
F3.2				Awareness of SPS	Interviewees describe and advise teachers to be more aware of SPS and highly sensitive people	I1, I3, I9	112

### 4.1 Self-definitions

During the interviews, the students appeared to be somewhat familiar with the construct of SPS (six out of nine). Only three of them (I1, I4, I9) claimed to be particularly aware of what SPS meant in their life. Notwithstanding this, while talking about their university experiences—and without any pressure, as we did not ask them to define themselves in any specific way (see Table A1)—they frequently relied on accounts in which they defined or labelled themselves as anxious (I2, I3, I6, I8, I9), shy (I1, I2, I3), or perfectionistic^1^ (I2, I9) (see Table 2).

These characteristics are consistent with SPS traits (Aron, 1996; Aron and Aron, 1997; Eşkisu et al., 2022). This excerpt pertains to being an introvert and anxious:

“I’ve always given more importance for me being shy. I’ve always connected it a lot, so there’s like, let’s say, it takes me a while to warm up to people; I take my time with it, um, but it’s very easy that, let’s say, when I find myself trusting someone, then I also show this side of me that is maybe very prone to getting anxious." (I2)

The tendency to rely on more familiar categories of shyness and social anxiety seemed to be quite common among our sample of students. Another category they often referred to was perfectionism, or “the tendency to set and pursue unrealistically high goals, strive for flawlessness, set excessively high standards for performance, and overly critically evaluate oneself” (Workye et al., 2023, p. 2). For instance:

“maybe the fact of being so … tendency to perfectionism, as to I don’t know if I have a certain amount of time I dedicate entirely to because I would like to succeed to, to do well, then after I realize that I do more than needed.” (I2)

In this case, the category of perfectionism was used to self-define the student while at the same time justifying herself for her excessive involvement and over-execution in academic achievement.

### 4.2 University experience

During data analysis, three themes were extrapolated that we found connected to our interviewees’ broader university experiences. They are: (1) *study approach*, which refers to emotions and behaviors that students narrate in reference to their relationship with studying (e.g., study strategies, preparation for exams); (2) *classroom experience*, which includes all those narratives referring to students’ experiences during (online and in-person) lessons; and (3) *physical, emotional, and cognitive states during and after exams (or thesis discussion)*, which includes the emotional, cognitive, and behavioral responses that occurred before, during, and after taking exams (or defending their thesis). Table 3 displays these themes and subthemes.

#### 4.2.1 Study approach

As illustrated in Table 3, this theme was divided into three subthemes: (1) *strategies for organizing studies*; (2) *high school/university performance*; and (3) *university career blocks*. In terms of “strategies for organizing studies,” participants who identified themselves as high-achieving university students (I1, I2, I3, I4, I5, I6, I7, I9) claimed to be supported by specific strategies (I3, I4, I5, I6, I8, I9). Indeed, they reported placing a high importance on arranging their study material, allotting their time, and planning their activities with timetables and schemes. For instance:

“My method to manage anxiety and stress is organization. So, usually when I know that I have to take some exam, when we start, I start to gather all materials, divide them, and organize tables.” (I9)

These strategies appeared linked to their need to manage their anxiety that overall accompanied their academic experience. Studying was narratively constructed as particularly demanding and tiring by most interviewees (I2, I3, I7, I8, I9), and the energies invested were often perceived—frequently, afterwards—as excessive compared to what was required or necessary (I2, I3). At the same time, however, their efforts appeared to be effective, as the majority of our sample reported highly successful academic performances (I1, I2, I3, I4, I5, I6, I7, I9). Interestingly, however, most admitted their success only indirectly, like this interviewee:

“Even more so, people would say to me, ‘why are you always complaining? Why are you like this?’ … about this and that, up and down, if in the end you always get 30s, 29s, 28s, or whatever.” (I6)

The same interviewee relied on an indirect phrasal construction—by voicing others’ complaints about her anxious attitude and constant self-undervaluation—to specify that she usually received the highest grades (the maximum grade in Italy is 30). While their successful academic experiences were modestly recalled, negative experiences seemed to be more easily and directly accessed. In the narratives of our interviewees, in fact, blocks affecting university choices frequently emerged. Students generally reported tension and anxiety during critical moments of their academic experience (I1, I4, I6), mainly linked to their choice of supervisor for their thesis (I4, I6). For example:

“I didn't have a great experience with my undergraduate thesis. I really liked the topic, I really enjoyed the research we did, but I didn't handle the whole period well because my professor wasn't guiding me properly. I tried to do some things the best I could, but I didn't know if they were actually done well. [...] What held me back was that it wasn't something that depended on me, so the fact that I couldn't achieve what I was working so hard to achieve made the experience difficult. There was a moment when I thought I couldn't take it anymore and just wanted to be done. The submission of the thesis was more of a relief than a satisfaction at that point.” (I1)

#### 4.2.2 Classroom experiences

Regarding university experiences during lectures, two subthemes emerged from the analysis of the interviews: (1) *online class experiences* and (2) *anxiety in speaking during lectures* (see Table 3). Regarding online classes—taken during COVID-19 lockdowns—eight out of nine students stated that online delivery was “distracting” (I2, I3, I5, I6, I7, I8, 19). They reported being exposed to stimuli of various kinds, whose management hindered their attention; for instance, using a microphone, chat, and camera could be distracting. A lack of interaction with classmates and teachers was described as a further obstacle to involvement and motivation. In the experiences of the interviewees, boredom, a sense of detachment, and unreality concerning surrounding environments was present, similar to their peers’ experiences (e.g., Ghislieri et al., 2023; Riboldi et al., 2023). However, some of the participants expressed their preference for online classes (I3, I4, I6), as these appeared to facilitate the management of emotions, including anxiety. Still, the possibility of turning off the camera or formulating responses in chat boxes, rather than exposing oneself in person, were considered sources of “protection” for the SPS students faced with the discomfort of public exposure. Instead, regardless of the class format—be it online or face-to-face—anxiety about public speaking therefore emerged as a particularly recurrent theme in the narratives of our interviewees (I1, I3, I4, I6, I7), suggesting that for highly sensitive students, anxiety is deeply rooted in classroom interaction. For instance:

“I was less anxious about going there, seeing what it's like, etcetera, because being at home I only had to experience the anxiety of intervening in class; it was always there, anyway.” (I4)

#### 4.2.3 Physical, emotional, and cognitive states during and after exams (or thesis discussion)

During interviews, the participants took a great number of turns (see Table 3) to elaborate what they encountered before, during, and after an exam—or thesis discussion (generally, six out of eight interviewees expressed their views about thesis discussions). In this respect, five subthemes emerged from our thematic analysis: (1) *preference for written exams*; (2) *anxiety during exams*; (3) *physical symptoms in the post-exam (or post-thesis discussion) phase*; (4) *emotional experiences in the post-exam (or post-thesis discussion) phase*; and (5) *cognitive-behavioral strategies in the post-exam (or post-thesis discussion) phase* (see Table 3).

Most interviewees expressed their preference for written exams (I1, I2, I3, I4, I6, I7) due to the relatively lower amount of (internal and external) stimuli they had to process from less interaction with teachers, no peers observing, more time to reflect, and possibilities to manage anxiety without affecting performance. For instance:

“If the exam is written, I'm always more relaxed. [...] On the other hand, during a written exam, since I only have to interact with the paper or, well, with the computer now, I feel less anxious because I know I can take my time to think and reason. I handle it better, so I feel different, depending on whether it's written or oral." (I1)

Instead, regarding oral exams, the interviewees reported major anxiety. For example:

“If the exam is oral, instead, I feel more anxious, often related to the fear of maybe not knowing the answer to what I'm asked and thus making a fool of myself in front of the professor. In reality, I know it's not like that because … because it isn't. It can happen that you don't know something or don't remember it well, but in my head, everything is amplified. It seems to me that if something goes wrong, it is perceived as a bigger deal than it actually is. So, this changes things for me.” (I1)

Overall, the participants reported being anxious about either the possibility of failing the test—not only concerning the grade itself, but also the negative impact of a possible poor performance on their self-image—and the fear of not being able to correctly explain what they had learned. They seemed to be particularly sensitive about the presence of others (I1, I2, I3, I4, I6, I7), by whom they often felt judged or in awe of, with only one exception (I9), where anxiety was present but not described as debilitating—the high emotional activation that accompanies the exam experience was generally perceived as an “obstacle” to learning, in some cases creating an emotional and behavioral block. Anxiety was also related to the use of technology during online exams (I2, I3, I4, I6, I8, I9), where students at the same time had to perform and control their online connection, like the possibility that their connection might drop or that something might go wrong on a technical level, which represented a source of great concern. These high levels of anxiety created real blocks that could threaten their academic achievements (I4, I5, I7, I8, I9).

Regarding post-exam or post-thesis discussion phases, students’ narratives revealed a range of physical symptoms, emotional states, and cognitive-behavioral strategies used to counterbalance over-arousal (see Table 3). Symptoms reported by students included physical (I2, I3, I4, I6, I9) and mental (I2, I3, I4, I5, I8) tiredness, headaches (I5, I6), leg problems (I3, I8), neck pain (I8), digestive problems (I5), hair loss (I9), and fever (I7). For instance, in the post-exam phase, physical symptoms could include exhaustion:

“I’m drained, completely drained. [...] This year I had an exam on March 29, an oral exam, and I, I went to bed, and I was there facing the void. I didn't even have the energy to eat. I ate late at night. I felt completely emptied, even emotionally. Suffice to say, I didn’t have anything left—not even the joy of saying that at least I did it.” (I2)

At the same time, like in the previous case, physical symptoms were accompanied by intense emotional experiences. They could be experiences of relief (I1, I3, I4, I5, I6, I7, I8, I9), or contentment and satisfaction (I1, I9). However, we collected no shortage of stories about feelings of disappointment or guilt (I1), anger (I9), and sadness (I1) when exams failed or could have gone better. These emotions were often accompanied by narratives related to cognitive and behavioral strategies used in the post-exam phase—narrated as either functional or unavoidable but nonfunctional—like brooding over one’s presentation for the exam (I1). In most cases, these strategies were remembered as aimed at restoring post-exam states of well-being through leisure activities (I1, I2, I4, I5, I6, I8) or avoidance (I5, I9), like:

“Then here you go for a walk, an aperitif, and then you also release a lot of tension, by also doing something that makes you feel good, no?” (I6)

### 4.3 Peer relationships

During data analysis, peer relationship issues emerged and were coded by means of three suthemes: 1) *peer relationship quality*, in which interviewees qualified their interactions with fellow students; 2) *barrier conditions*, in which they listed the conditions that hindered a good-enough relationship with peers; and 3) *favorable conditions*, in which interviewees explained the conditions that favored good relationships. Table 4 illustrates these results.

#### 4.3.1 Peer relationship quality

The relational context with fellow students was particularly important for our interviewees. Their narratives described several experiences of positive peer relationships, where students felt comfortable and supported by their friends at university (I1, I3, I6, I7, I9). For example:

“In general, I have always gotten along very well. I have never encountered people with whom I argued or intensely debated. On the contrary, there has always been a, a fairly mutual, helpful atmosphere, especially when needed." (I9)

At the same time, there were also relationship difficulties reported, especially when highly sensitive students faced new friends or acquaintances (I1, I2, I3, I6, I7). In one of the few narratives of our dataset in which interviewees explicitly referred to SPS, an interviewee reflected on peer relationships and “sensitivity,” oscillating between acknowledging the limitations and downplaying their impact on her social interactions:

“Maybe about some relationships with friends I have created, maybe high sensitivity^2^ has blocked me a little, held me back in going very deep in making myself known or in knowing others. However, overall, I don't think that it hindered me so much.” (I3)

#### 4.3.2 Barrier conditions

Our thematic analysis also showed that, among the factors hindering the development of healthy and functional peer relationships, emerged the fear of being judged (I3, I4, I6, I, I8, I9), which sometimes could be so pervasive that it drove participants to self-exclusion (I9), like:

“When there are situations that may be slightly ambiguous, I tend to create more problems for myself than might not actually exist and to feel more anxious. So, I start thinking, ‘maybe it’s not worth continuing to seek out the group because I don't feel like an integral part of it.’ Or maybe I said or did something that might have annoyed someone, so sometimes I exclude myself for this reason to avoid feeling like an extra.” (I9)

One of the loci in which this difficulty could take place was, particularly, oral exams (see above), where highly sensitive students seemed to suffer the most from others’ reactions. In the following excerpt, the interviewee shared a fiction, i.e., a narrative in which the teller explored an “evocation of imaginary characters, acts, or scenarios as test situations for the problem at hand” (Fasulo and Zucchermaglio, 2008, p. 355) to try to convey her emotional activation when peers might laugh at her possibly poor performance:

“Anyway, people are there, um, not all of them, but there are those who still stand there and say … they're like, if you make a mistake … they'll giggle.” (I4)

#### 4.3.3 Favorable conditions

Conversely, the interviewees recognized and included a whole range of factors that facilitated their social relationships with colleagues, including working in small groups (I1, I3, I4, I8, I9). This method was then perceived as a resource not only from an instrumental point of view, i.e., for study and learning, but also as a resource of emotional support. Interviewees particularly favored peer contexts where the climate was relaxed and in which they could discuss topics of common interest, mostly topics of study (I1, I3, I5), like:

“I have to create my own right environment a bit, um, so I like to be with people who, as well, they do the same things, so they too, anyway, they too study, so I can compare myself to [them] with which I can, maybe, um, even just taking inspiration from, as when you have a doubt, or in any case compare to, and or even keep in line for a moment, and in fact I was affected by this because the first semester we did with COVID^3^ I found it much more difficult to, to study on my own, and so this, yes … that is the environment … is very important to me.” (I5)

### 4.4 Student–teacher relationships

Regarding student-teacher relationships, three subthemes emerged: (1) *student–teacher relationship quality*, in which interviewees qualified their interaction with university educators; (2) *barrier conditions*, in which students described conditions that hindered a good-enough relationship with teachers; and (3) *favorable conditions*, in which students instead recalled either actual or potential conditions that favored good relationships with teachers. See Table 5.

#### 4.4.1 Student–teacher relationship quality

The interviewees described their relationships with teachers as generally “tense” (I1, I3, I5, I6, I7). The students reported feeling particularly afraid of teachers’ judgment, and students struggled to create healthy and functional relationships with them. For instance:

“I'm definitely struggling a bit, that is, I also see, um, I notice that really maybe, um, I compare [myself] to my colleagues, so, with a bit of admiration, from that point of view because I, from that point of view there … I always see the teacher as a teacher, as a person with whom … that is, when I speak to them, I am perhaps a bit intimidated and so … um ... going there and talking to them or, I don't know, chatting at the bar, I always keep to myself. I'm not the typical person who builds a relationship with teachers; maybe those two with whom I … whom I admire so much that it happened with me to ask them, I don’t know, about the internship or whatnot. That is, then when I meet them, I feel happy, but in reality … that is, for me, with the professor, with the teacher, I really struggle. I'm always a little intimidated, but not because they maybe … um … they perhaps are very calm people.” (I7)

Still, “good” interactions could occur (I2, I4, I5, I6, I7, I8), as in this extract:

“But unexpectedly, the last professor with whom I took my last exam during the winter session actually made a big difference. I was very apprehensive about him because I thought he was one of those professors who couldn't put you at ease and would instead make you even more anxious. But I entered the online exam room, and the first thing he noticed was that I was nervous. The first thing he said to me was, 'Stay calm.' When he said that, I felt completely at ease. I said to myself, 'Okay.' So, yes, it happened unexpectedly, and it actually helped me because the exam went very well.” (I1)

Globally, good relationships are characterized by teachers’ positive attitudes towards students’ emotional experiences, and teachers can become a source of support and reassurance who consequently enhance students’ academic performance.

#### 4.4.2 Barrier conditions

When talking about their difficult relationships with teachers, interviewees described their “struggles” (I1, I3, I5, I6, I7), namely their personal difficulties in unblocking themselves from the fear of being judged. Particularly, this internal block—connected with social anxiety—could be so rooted as to cause them to have minimum relationships, independent of teachers’ attitudes and/or calmness. For instance:

“In the last 2 years, I realized I started to unblock myself a little, because I realize it's *my* block. But … that is, I live it like this, I don't have any kind of relationship. That is, I interact in the corridors, I consider myself a polite person, but one that doesn't get on with people so easily.” (I7)

#### 4.4.3 Favorable conditions

When asked to reflect on what might improve the interviewees’ relationships with teachers, the interviewees expressed their need to be understood as highly sensitive individuals, especially concerning their anxiety and/or fear of exposure in public (I1, I3, I9), for instance:

“A non-judgmental environment, and a serene calm environment: these could help myself open a bit.” (I3)

The interviewees emphasized how it was important for them to receive clear feedback on their performances, not only in terms of learning, but more broadly in terms of social interactions. Indeed, this quest for reassurance undergirded interviewees’ need for emotional support (I1, I2, I3, I4, I5, I6, I7, I8, I9). For example, pertaining to exams, the following scenario well illustrates a student’s preferred favorable conditions, directly pertaining to an imaginary university educator:

“Start with a few words, if not of comfort, but a few words that break the ice a little. So don't, um, [say] ‘Sit. Name. Surname. Registration number. Well? Let's get started.’ But I don't know, simply, ‘Sit down. How are you? Are you nervous?’ Just a few words on your status, maybe making a joke together [is preferred]. In my opinion, it helps a lot to … or starting with a topic of your choice. In my opinion, it can put you in, in a sort of comfort zone, because the topic of your choice … it is assumed to be a topic you’re quite sure about. So, maybe the first obstacle is surmounted, let's say, of the exam. [...] I had exams where professors were impassive and where maybe you don't know if you're saying the right thing.” (I9)

## 5 Discussion

To our knowledge, the present study was among the first to investigate the narratives of highly sensitive people regarding their academic experiences. Although Aron (1996, 2002) extensively relied on interviews to formulate the initial SPS construct (Aron and Aron, 1997), the related scholarship over the past 20 years (with a few exceptions, such as Lindsay, 2017; Black and Kern, 2020; Roth et al., 2023) has largely neglected narratives as loci where highly sensitive individuals can make sense of their characteristics and socialization processes. This oversight is surprising and sad, as narratives are the “socially organized telling of temporally ordered past, present, or future events from a particular point of view” (Ochs and Taylor, 1992, p. 32). As dynamic processes involving the construction and negotiation of meanings through personal experiences, narratives can go far beyond merely recounting past events to actively shaping identities and making sense of lived experiences (Labov and Waletzky, 1967; Ochs and Capps, 1996, 2009). In socialization terms, narratives are widely considered eminent vehicles of socialization, as narratives are integral to any socialization process, as demonstrated by the literature on communities of practice and their narrative repertoires (Orr, 1991; Lave and Wenger, 1991; Wenger, 1999).

In this respect, our preliminary results help to unveil the power of students’ narratives to understand the complex academic socialization process in which they are embedded. The students did so by envisioning in detail their physical, emotional, and cognitive states related to their minute academic experiences in classrooms, before, during, and after exams, and with peers and educators.

Corroborating Liss et al. (2005, 2008) and Yano et al. (2021), participants in our study described themselves as high academic performers. However, they also considered this achievement as particularly stressful (e.g., May and Pitman, 2023), reproaching themselves for their over-effort at the cost of significant stress, as similarly found by Gerstenberg (2012) in his experimental tests with SPS students. This was consistent with the students’ self-definitions of anxious, shy, and perfectionist. Considering Smith et al.’s (2016) definition of perfectionism, in our dataset, perfectionism was used as a self-critical tool that implied negative student comments on their own (academic) conduct (Roth et al., 2023). This has been shown to have the strongest association with distressing emotions among university students (Casale et al., 2020; Workye et al., 2023), which was also the case for some of our participants. In this respect, our results—albeit preliminary—corroborate previous qualitative research (Black and Kern, 2020; Roth et al., 2023), showing that anxiety can appear as a pervasive transversal experience that affects students’ overall academic experiences and relationships. Cohering with Liss et al. (2005, 2008) and Jagiellowicz et al. (2016), in our dataset, anxiety was narratively constructed as something that could even block students in their career and hinder them from delving deeper in relationships. In this respect, our results seemed to stress that the students assumed university socialization was an individual responsibility, and they blamed themselves for their difficulties with peers and teachers. In this sense, it seemed that most interviewees likely aligned with the profile of highly sensitive individuals called “orchids” (Lionetti et al., 2018), as they reported higher emotional reactivity and lower extraversion.

Regarding their relationships with colleagues, some students recalled having “social blocks” with colleagues, avoiding intimate interactions, and fearing judgment, particularly during exams. Even though they felt blamed for being considered “avoidant” and felt risk from isolating themselves—preferring to be with a few people only—they expressed a preference for small group interactions within positive and inclusive environments. In our dataset, however, peers were frequently (narratively) constructed in terms of comparison, either with stressful notes—pointing to what they did best or did without significant effort and as sources of anxiety—and/or with reassuring notes—like when labeled as sources of inspiration. Our results suggest that, when peer interaction works well, highly sensitive students find support and comfort, particularly when studying.

Regarding interactions with university educators, in our dataset the interviewees described both formal and informal interactions. In both contexts, however, most students reported struggling to build constructive relationships due to their fear of judgment, over-idealization, and introversion, hindering them from developing more meaningful relationships.

In terms of understanding what helped and hindered the highly sensitive students during academic socialization, our preliminary results showed that they could benefit from several specific resources, such as inclusive and non-judgmental, small social learning environments, detailed study plans, wise time management, small group interactions, online lessons and written exams, and/or in-person oral exams with limited attendees. These findings—even if circumstantial—partially corroborate research on online communication among highly sensitive people (Bordarie et al., 2022; Pérez-Chacón et al., 2021; Iumura, 2022), particularly the mediation role of online communication in interpersonal relationships. While Valojää’s (2015) results suggest that “the Internet as an environment enabled the HSPs to be less shy and more sociable than they were in face-to-face interaction” (p. 2), our findings indicate that online communication did not entirely substitute or explain students’ preferences. Instead, preferences depended on specific situations and the requirements that the highly sensitive students perceived.

### 5.1 Strengths, limitations, and future research

This study had several strengths and limitations worth noting. Among the strengths, this research addressed highly sensitive university students’ participation in a university system, a topic area with very limited literature. Specifically, our sample included not only psychology students—who are the most commonly studied group in the SPS and ES literatures—but also students of different subjects. While taking an approach different from the prevalent quantitative approach that often focuses on clinical conditions and (mal)adaptation, we originally focused on the students’ broader academic experiences and resources. By analyzing students’ narratives on academic socialization, the study highlights their reflexivity regarding their university experience, using their words to shed light on this complex, bidirectional, and ongoing process in which institutions, formal and informal networks, opportunities, and individuals can make a significant difference. Our methodological approach is notable for its originality, as there are few qualitative studies in the SPS and ES literatures (see for an extensive review), (see, for instance, Lindsay, 2017; Black and Kern, 2020; Roxburgh, 2022; Roth et al., 2023). However, we recognized several limitations that impact our results’ generalizability. First, this study was preliminary, and its sample size was small and limited to students attending two Italian universities only. Thus, findings cannot be taken as representing the entire Italian university population with high SPS. We also did not include individuals who had dropped out university, raising issues of actual representation of academic difficulties and setbacks. Representativeness issues also apply to the Italian context itself. Compared to other countries like Germany, the United Kingdom, and the USA, where there is greater awareness and cultural sensitivity to SPS-related issues, the debate around SPS in Italy is less developed. Finally, we recognized the potential impact of self-selection bias. To be included in this study, we required participants to use Aron’s (1996) 20-item self-report test, which was administered independently of the researchers and without academic or clinical supervision. This self-assessment could have affected the results’ accuracy.

By accounting for the aforementioned study limitations, future research should delve deeper into the academic experiences of highly sensitive students, not only as frequent social science research participants. To capture the richness and complexity of their experiences, more in-depth qualitative research is needed, such as observational research, in-depth ethnographic interviews, and/or narrative analyses. To better focus on outcomes of academic socialization processes, longitudinal approaches could also produce useful insights into the long-term effects of SPS on academic socialization and adaptation. Also, expanding the study sample to include students from a more diverse range of universities and cultural backgrounds would enhance the generalizability of findings. Additionally, future studies could employ more rigorous diagnostic methods for identifying highly sensitive university students, such as clinical assessments conducted by trained professionals.

## 6 Conclusion and practical implications

Since academic socialization is a biunivocal process that requires reciprocal adaptation from students and universities (see Farnese et al., 2022), this study might lead to productive conversations about the adaptation of this specific minority population and their relationships with academic institutions. This is particularly vital given that secondary socialization experiences (e.g., school and other extracurricular experiences) exert substantial impacts not only during childhood and adolescence (e.g., Rubin et al., 2011; Cecalupo et al., 2022; Marini et al., 2023) but also and more broadly on individuals’ overall adaptation and responses to developmental challenges throughout entire life cycles (e.g., Zandvliet et al., 2014; Wentzel, 2016; Livi and Rullo, 2017; Ryan and Deci, 2020).

Overall, we hope this study contributes to a new chapter in the fellowship of SPS in real-life settings and socialization experiences, offering several practical implications for students, university educators, and institutions. For highly sensitive students, including doctoral candidates and researchers, this study recognized the unique resources and challenges associated with SPS within university settings. In this light, it can be crucial for them to identify and develop effective coping strategies at critical junctures in their academic careers, like during exams, transitions, presentations, career decision-making points, and unexpected setbacks, to monitor themselves and prevent significant psychosomatic symptoms that our preliminary results illustrate were fairly common in this population. For university educators, it is essential to become aware and recognize that a segment of the student population has a distinct profile that requires specific attention. In this respect, this study might help in constructing and delivering specific professional trainings for university teachers focused on SPS and academic socialization, thereby fostering strategies that can contribute to more inclusive educational settings and teaching techniques, such as classroom organization, small group work techniques, and strategies to help manage students’ anxiety. At the institutional level, our study advocates for the development of inclusive strategies that promote effective socialization for all students, taking into consideration their voices and needs and fostering new adaptive ways to address university stress, which is alarmingly growing worldwide (Beiter et al., 2015). Developing awareness campaigns on neurodiversity and minority stress, expanding mental health consultancy services to tackle university stress, and implementing formal and informal approaches to enhance students’ well-being can be effective for entire student bodies, including those highly sensitive students.

## Data Availability

The raw data supporting the conclusions of this article will be made available by the authors, without undue reservation.

## Footnotes

^1^We separately coded their qualifications of “anxious” and “perfectionist” since recent studies on students’ academic achievement considered these two related states as separate (Workye et al., 2023).

^2^High sensitivity, i.e., *alta sensibilità* in Italian, is the most widely used term for SPS in informal conversation.

^3^In informal Italian conversation, the term “COVID” connotes the period of lockdown due to COVID-19 restrictions in which universities converted to online communication, delivering online lectures and exams.

## Appendix

**Table A1:** Semi-structured interview—list of questions.

Have you ever heard of high sensitivity in your course? Are you an off-site student? Do you attend classes? If so, do you prefer to attend online or in person? How do you get on with your fellow students? Do you think being highly sensitive has affected your relationships at university? How do you manage exams and studying in relation to high sensitivity? Do you prefer oral or written exams? Online or in-presence? How do you manage your relationships with teachers? What advice would you give to a teacher who is faced with a highly sensitive student?

## References

[ref2] AlmaLaurea (2023). XXV Indagine Profilo dei Laureati 2022. Rapporto 2023. Available at: https://www.almalaurea.it/i-dati/le-nostre-indagini/profilo-dei-laureati (Accessed May 25, 2024)

[ref3] Aron E. N. (1996). The highly sensitive person. How to thrive when the world overwhelms you. New York: Harmony/Rodale.

[ref4] Aron E. N. (1999). The highly sensitive Person’s workbook. The practical guide for highly sensitive people and HSP support groups. New York: Harmony/Rodale.

[ref5] Aron E. N. (2002). The highly sensitive child. New York: Harmony.

[ref6] Aron E. N. Aron A. (1997). Sensory-processing sensitivity and its relation to introversion and emotionality. J. Pers. Soc. Psychol. 73, 345–368. doi: 10.1037/0022-3514.73.2.345, PMID: 9248053

[ref7] Aron E. N. Aron A. Davies K. M. (2005). Adult shyness: the interaction of temperamental sensitivity and an adverse childhood environment. Personal. Soc. Psychol. Bull. 31, 181–197. doi: 10.1177/0146167204271419, PMID: 15619591

[ref8] Aschieri F. Fantini F. Antonelli A. Van Ryzin M. Smith J. D. (2024). Therapeutic assessment in a university counseling center: a replicated single-case time-series pilot study. J. Pers. Assess.. 106, 546–557. doi: 10.1080/00223891.2023.2296065 38180034

[ref9] Beiter R. Nash R. McCrady M. Rhoades D. Linscomb M. Clarahan M.. (2015). The prevalence and correlates of depression, anxiety, and stress in a sample of college students. J. Affect. Disord. 173, 90–96. doi: 10.1016/j.jad.2014.10.054, PMID: 25462401

[ref10] Black B. A. Kern M. L. (2020). A qualitative exploration of individual differences in wellbeing for highly sensitive individuals. Palgrave Commun. 6, 1–11. doi: 10.1057/s41599-020-0482-8

[ref11] Bordarie J. Aguerre C. Bolteau L. (2022). A longitudinal approach of lockdown effects on quality of life and the expression of anxiety-depressive disorders according to sensory processing sensitivity. Psychol. Health Med. 27, 2288–2299. doi: 10.1080/13548506.2021.1968012, PMID: 34407714

[ref12] Braun V. Clarke V. (2006). Using thematic analysis in psychology. Qual. Res. Psychol. 3, 77–101. doi: 10.1191/1478088706qp063oa

[ref13] Braun V. Clarke V. (2022). Toward good practice in thematic analysis: avoiding common problems and be(com)ing a knowing researcher. Int. J. Transgender Health 24, 1–6. doi: 10.1080/26895269.2022.2129597, PMID: 36713144 PMC9879167

[ref15] Casale S. Fioravanti G. Rugai L. Flett G. L. Hewitt P. L. (2020). What lies beyond the superordinate trait perfectionism factors? The perfectionistic self-presentation and perfectionism cognitions inventory versus the big three perfectionism scale in predicting depression and social anxiety. J. Pers. Assess. 102, 370–379. doi: 10.1080/00223891.2019.1573429, PMID: 30907635

[ref16] Cecalupo A. Marini M. Scarci F. Livi S. (2022). Individual strivings in social comparison processes: achievement motivation goals in the big-fish-little-pond effect. Front. Psychol. 13:677997. doi: 10.3389/fpsyg.2022.677997, PMID: 35519645 PMC9062594

[ref17] Eşkisu M. Ağırkan M. Çelik O. Yalçın R. Ü. Haspolat N. K. (2022). Do the highly sensitive people tend to have psychological problems because of low emotion regulation and dysfunctional attitudes? J. Ration. Emot. Cogn. Behav. Ther. 40, 683–706. doi: 10.1007/s10942-021-00436-w

[ref18] European Commission (2017) Communication from the commission to the European parliament, the council, the European economic and social committee and the committee of the regions on a renewed EU agenda for higher education. COM/2017/0247 final, Document 52017DC0247

[ref19] Farnese M. L. Spagnoli P. Livi S. (2022). Undergraduates’ academic socialization. A cross-time analysis. Br. J. Educ. Psychol. 92, 1239–1255. doi: 10.1111/bjep.12497, PMID: 35274739 PMC9790489

[ref003] Fasulo A. Zucchermaglio C. (2008). Narratives in the workplace: facts, fiction and canonicity. Text & Talk. 28, 351–376. doi: 10.1515/TEXT.2008.017

[ref20] Gerstenberg F. X. (2012). Sensory-processing sensitivity predicts performance on a visual search task followed by an increase in perceived stress. Personal. Individ. Differ. 53, 496–500. doi: 10.1016/j.paid.2012.04.019

[ref21] Ghislieri C. Sanseverino D. Dolce V. Spagnoli P. Manuti A. Ingusci E.. (2023). Emotional exhaustion and engagement in higher education students during a crisis, lessons learned from COVID-19 experience in Italian universities. Soc. Sci. 12:109. doi: 10.3390/socsci12020109

[ref23] Greven C. U. Lionetti F. Booth C. Aron E. N. Fox E. Schendan H. E.. (2019). Sensory processing sensitivity in the context of environmental sensitivity: a critical review and development of research agenda. Neurosci. Biobehav. Rev. 98, 287–305. doi: 10.1016/j.neubiorev.2019.01.009 30639671

[ref24] Hennink M. Kaiser B. N. (2022). Sample sizes for saturation in qualitative research: a systematic review of empirical tests. Soc. Sci. Med. 292:114523. doi: 10.1016/j.socscimed.2021.114523 34785096

[ref25] Jagiellowicz J. Aron A. Aron E. N. (2016). Relationship between the temperament trait of sensory processing sensitivity and emotional reactivity. Soc. Behav. Personal. Int. J. 44, 185–199. doi: 10.2224/sbp.2016.44.2.185

[ref26] Jagiellowicz J. Xu X. Aron A. Aron E. Cao G. Feng T.. (2011). The trait of sensory processing sensitivity and neural responses to changes in visual scenes. Soc. Cogn. Affect. Neurosci. 6, 38–47. doi: 10.1093/scan/nsq001 20203139 PMC3023077

[ref27] Labov W. Waletzky J. (1967). Narrative analysis: oral versions of personal experience In: Essays on the verbal and visual arts. ed. Helm J. (Seattle and London: University of Washington Press). 3–38.

[ref28] Lave J. Wenger E. (1991). Situated learning: Legitimate peripheral participation: Cambridge University Press.

[ref29] Lindsay J. S. (2017). The Highly Sensitive Teacher: Sensory-Processing Sensitivity, Burnout, and Self-Efficacy in Urban Public School Teachers discussed at University of California Los Angeles.

[ref30] Lionetti F. Aron A. Aron E. N. Burns G. L. Jagiellowicz J. Pluess M. (2018). Dandelions, tulips and orchids: evidence for the existence of low-sensitive, medium-sensitive and high-sensitive individuals. Transl. Psychiatry 8:24. doi: 10.1038/s41398-017-0090-6, PMID: 29353876 PMC5802697

[ref31] Liss M. Mailloux J. Erchull M. J. (2008). The relationships between sensory processing sensitivity, alexithymia, autism, depression, and anxiety. Personal. Individ. Differ. 45, 255–259. doi: 10.1016/j.paid.2008.04.009

[ref32] Liss M. Timmel L. Baxley K. Killingsworth P. (2005). Sensory processing sensitivity and its relation to parental bonding, anxiety, and depression. Personal. Individ. Differ. 39, 1429–1439. doi: 10.1016/j.paid.2005.05.007

[ref33] Livi S. Rullo M. (2017). “Group processes” in The SAGE encyclopedia of theory in psychology. ed. Miller H., vol. 2 (Thousand Oaks, CA, USA: SAGE Publications Ltd.), 389–392.

[ref34] Marini M. Di Filippo G. Bonuomo M. Torregiani G. Livi S. (2023). Perceiving oneself to be integrated into the peer group: a protective factor against victimization in children with learning disabilities. Brain Sci. 13:263. doi: 10.3390/brainsci13020263, PMID: 36831805 PMC9954448

[ref35] May A. K. Pitman M. M. (2023). The association between sensory processing sensitivity, the five-factor model and university adjustment amongst south African university students. Curr. Psychol. 42, 7938–7952. doi: 10.1007/s12144-021-02035-5

[ref37] Ochs E. Capps L. (1996). Narrating the self. Annu. Rev. Anthropol. 25, 19–43. doi: 10.1146/annurev.anthro.25.1.19

[ref38] Ochs E. Capps L. (2009). Living narrative: creating lives in everyday storytelling. Cambridge, MA: Harvard University Press.

[ref40] Ochs E. Taylor C. (1992). “Science at Dinner”. In Text Context: Cross Disciplinary Perspectives on Language Study. eds. Kramsch C. McConnell-Ginet S.. Lexington, MA: D.C. Heath. 29–45.

[ref41] Orr J. (1991). Talking about machines. An ethnography of of a modern job. Ithaca and London: ILR press an imprint of cornell university press.

[ref42] Pagani V. (2020). Dare voce ai dati. L’analisi dei dati testuali nella ricerca educativa. Reggio Emilia: Edizioni Junior.

[ref43] Patton M. Q. (1990). Qualitative evaluation and research methods. 2nd ed. Angeles L.. Washington DC, and Toronto: Sage.

[ref44] Pérez-Chacón M. Chacón A. Borda-Mas M. Avargues-Navarro M. L. (2021). Sensory processing sensitivity and compassion satisfaction as risk/protective factors from burnout and compassion fatigue in healthcare and education professionals. Int. J. Environ. Res. Public Health 18:611. doi: 10.3390/ijerph18020611, PMID: 33445789 PMC7828252

[ref45] Pluess M. (2015). Individual differences in environmental sensitivity. Child Dev. Perspect. 9, 138–143. doi: 10.1111/cdep.12120

[ref46] Riboldi I. Capogrosso C. A. Piacenti S. Calabrese A. Lucini Paioni S. Bartoli F.. (2023). Mental health and COVID-19 in university students: findings from a qualitative, comparative study in Italy and the UK. Int. J. Environ. Res. Public Health 20:4071. doi: 10.3390/ijerph20054071, PMID: 36901083 PMC10001873

[ref045] Roxburgh E. C. (2022). “It’s Like Feeling and Experiencing Everything in HD”: It’s Like Feeling and Experiencing Everything in HD. The Humanistic Psychologist 51, 377–396. doi: 10.1037/hum0000297

[ref47] Roth M. Gubler D. A. Janelt T. Kolioutsis B. Troche S. D. (2023). On the feeling of being different–an interview study with people who define themselves as highly sensitive. PLoS One 18:e0283311. doi: 10.1371/journal.pone.0283311, PMID: 36930633 PMC10022759

[ref48] Rubaltelli E. Scrimin S. Moscardino U. Priolo G. Buodo G. (2018). Media exposure to terrorism and people's risk perception: the role of environmental sensitivity and psychophysiological response to stress. Br. J. Psychol. 109, 656–673. doi: 10.1111/bjop.12292, PMID: 29498033

[ref49] Rubin K. H. Bukowski W. M. Laursen B. (2011). Handbook of peer interactions, relationships, and groups. New York, NY: Guilford Press.

[ref50] Ryan R. M. Deci E. L. (2020). Intrinsic and extrinsic motivation from a self-determination theory perspective: definitions, theory, practices, and future directions. Contemp. Educ. Psychol. 61:101860. doi: 10.1016/j.cedpsych.2020.101860

[ref52] Tinto V. (1975). Dropout from higher education: a theoretical synthesis of recent research. Rev. Educ. Res. 45, 89–125. doi: 10.3102/00346543045001089

[ref54] Tinto V. (2006). Research and practice of student retention: what next? J. College Student Retent 8, 1–19. doi: 10.2190/4YNU-4TMB-22DJ-AN4W

[ref001] Tinto V. (2022). Exploring the character of student persistence in high education: The impact of perception, motivation, and engagement. In: The handbook of research on student engagement (2nd ed.). eds. Reschly A. L Christenson S. L. New York, NY: Springer.

[ref55] Valojää A. (2015). The effect of sensory-processing sensitivity on social preference for online interaction and associated outcome on psychological well being. Master's thesis.

[ref002] Weidman J. C. (2006). Socialization of Students in Higher Education: Organizational Perspectives in The Sage Handbook for Research in Education: Engaging Ideas and Enriching Inquiry. eds. Conrad C. C. Serlin R. C. 2nd ed (Thousand Oaks, CA: Sage), 253–262.

[ref56] Weidman J. C. DeAngelo L. Bethea K. A. (2014). Understanding student identity from a socialization perspective. N. Dir. High. Educ. 2014, 43–51. doi: 10.1002/he.20094

[ref57] Wenger E. (1999). Communities of practice: learning, meaning and identity. Cambridge, MA: Cambridge University Press.

[ref58] Wentzel K. R. (2016). “Teacher-student relationships” in Handbook of motivation at school. eds. Wentzel R. Miele D.. 2nd ed (New York and London: Routledge), 301–322.

[ref59] Workye R. Shephard A. Alexander S. M. Cribbie R. A. Flett G. L. Mackinnon S. P. (2023). Perfectionism, anxiety sensitivity, and negative reactions following a failed statistics test: a vulnerability–stress model. Scholarsh. Teach. Learn. Psychol. doi: 10.1037/stl0000363

[ref60] Yano K. Kase T. Oishi K. (2021). Sensory processing sensitivity moderates the relationship between life skills and depressive tendencies in university students. Jpn. Psychol. Res. 63, 152–163. doi: 10.1111/jpr.12289

[ref61] Zandvliet D. Den Brok P. Mainhard T. (2014). Interpersonal relationships in education: from theory to practice. Rotterdam, Boston and Taipei: Springer.

